# Extracellular vesicle mimics made from iPS cell-derived mesenchymal stem cells improve the treatment of metastatic prostate cancer

**DOI:** 10.1186/s13287-020-02097-5

**Published:** 2021-01-07

**Authors:** Qingguo Zhao, Bo Hai, Jack Kelly, Samuel Wu, Fei Liu

**Affiliations:** Institute for Regenerative Medicine, Molecular and Cellular Medicine Department, College of Medicine, Texas A&M University Health Science Center, College Station, TX 77843 USA

**Keywords:** Mesenchymal stem cells, Induced pluripotent stem cells, Prostate cancer, Metastasis, Extracellular vesicle mimics, Nanovesicles, Targeted cancer therapy

## Abstract

**Background:**

Extracellular vesicles (EVs) and their mimics from mesenchymal stem cells (MSCs) are promising drug carriers to improve cancer treatment, but their application is hindered by donor variations and expansion limitations of conventional tissue-derived MSCs. To circumvent these issues, we made EV-mimicking nanovesicles from standardized MSCs derived from human induced pluripotent stem cells (iPSCs) with a theoretically limitless expandability, and examined the targeting capacity of these nanovesicles to prostate cancer.

**Methods:**

Nanovesicles are made from intact iPSC-MSCs through serial extrusion. The selective uptake of fluorescently labeled nanovesicles by prostate cancer cells vs. non-tumor cells was examined with flow cytometry. For in vivo tracing, nanovesicles were labeled with fluorescent dye DiR or renilla luciferase. In mice carrying subcutaneous or bone metastatic PC3 prostate cancer, the biodistribution of systemically infused nanovesicles was examined with in vivo and ex vivo imaging of DiR and luminescent signals. A chemotherapeutic drug, docetaxel, was loaded into nanovesicles during extrusion. The cytotoxicities of nanovesicle-encapsulated docetaxel on docetaxel-sensitive and -resistant prostate cancer cells and non-tumor cells were examined in comparison with free docetaxel. Therapeutic effects of nanovesicle-encapsulated docetaxel were examined in mice carrying subcutaneous or bone metastatic prostate cancer by monitoring tumor growth in comparison with free docetaxel.

**Results:**

iPSC-MSC nanovesicles are more selectively taken up by prostate cancer cells vs. non-tumor cells in vitro compared with EVs, membrane-only EV-mimetic nanoghosts and liposomes, which is not affected by storage for up to 6 weeks. In mouse models of subcutaneous and bone metastatic PC3 prostate cancer, systemically infused nanovesicles accumulate in tumor regions with significantly higher selectivity than liposomes. The loading of docetaxel into nanovesicles was efficient and did not affect the selective uptake of nanovesicles by prostate cancer cells. The cytotoxicities of nanovesicle-encapsulated docetaxel are significantly stronger on docetaxel-resistant prostate cancer cells and weaker on non-tumor cells than free docetaxel. In mouse models of subcutaneous and bone metastatic prostate cancer, nanovesicle-encapsulated docetaxel significantly decreased the tumor growth and toxicity to white blood cells compared with free docetaxel.

**Conclusions:**

Our data indicate that EV-mimicking iPSC-MSC nanovesicles are promising to improve the treatment of metastatic prostate cancer.

## Background

Prostate cancer (PCa) is the second most frequent cancer in men and the fifth leading cause of death worldwide [1]. Skeletal metastases occur in more than 80% of cases of advanced-stage PCa and often become resistant to androgen deprivation therapy and cytotoxic chemotherapies [2]. Despite recent advancements of novel therapies, the relative 5-year survival rate for distant stage PCa is still < 30%. Nanomedicine of PCa is a promising direction, but mainstream synthesized nanoparticles cannot efficiently deliver anti-cancer agents into metastatic PCa due to the lack of active targeting capacity and the limited uptake of these nanoparticles by PCa cells [3, 4].

Natural extracellular vesicles (EVs) and EV-mimetic nanovesicles (NVs) are taken up by cancer cells more efficiently than synthetic nanoparticles through active endocytosis pathways such as enhanced macropinocytosis (cell drinking) [5]. Moreover, EVs and NVs are favorable drug carriers to overcome the multidrug resistance of cancer cells caused by the increase of drug efflux via hyperactive membrane transporters. Compared with free drugs taken up mainly by passive diffusion through the cell membrane, drugs carried by EVs and NVs are released distal to the cell membrane and therefore less likely to be extruded by membrane transporters [5]. Consequently, the encapsulation of chemotherapy drugs with EVs or NVs dramatically increased drug accumulation and cytotoxicity in multidrug-resistant renal cancer cells (up to 50-fold compared with free drugs) [6], and improved therapeutic effects in animal models of colorectal cancer [7].

Mesenchymal stem cells (MSCs) interact with PCa cells through multiple surface molecules, and EV-mimicking nanoghosts made from bone marrow (BM) MSC membranes showed active PCa-targeting capacity in a mouse model of subcutaneous PCa [8, 9]. However, nanoghosts cannot deliver cytoplasmic components such as proteins and nucleic acids expressed by MSCs. Moreover, MSCs isolated from tissues such as bone marrow have high donor variations and limited expandability and lose some important biological functions after prolonged expansion [10–14]. Therefore, it is challenging to use tissue-derived MSCs as reliable sources for the large amounts of standardized EV mimics required for further research and future clinical application [15].

To address the cell source issues, we established MSCs from induced pluripotent stem cells (iPSCs) with a theoretically limitless expandability [16]. These iPSC-derived MSCs (iPSC-MSCs) are highly consistent in the homing capacities to various cancers and the expression of surface molecules related to cancer targeting [16–18]. The osteogenic potentials of our iPSC-MSCs are superior to BM-MSCs [16, 19], while the immunomodulatory capacities are comparable between these two types of MSCs [20, 21]. We have prepared nanoghosts from membrane-only ghost iPSC-MSCs and nanovesicles from intact iPSC-MSCs respectively by serial extrusions [18]. Compared with nanoghosts, iPSC-MSC nanovesicles showed a much higher production yield and a smaller size that is related to better tumor penetration [18]. We report here that iPSC-MSC nanovesicles are capable of targeting human PCa xenografts in mouse models of subcutaneous and bone metastatic PCa, and significantly enhanced therapeutic effects of docetaxel, a PCa chemotherapy drug, with decreased toxicity to white blood cells.

## Methods

### Cells

The iPSC-MSCs were recovered from frozen vials of a cell bank that has been characterized extensively including the trilineage differentiation in comparison with bone marrow MSCs from multiple donors [16, 19]. These cells were plated at a density of 500 cells per square centimeter of growth area in 17% FBS αMEM medium at 37 °C and 5% CO_2_ and passaged upon 70–80% confluence. The iPSC-MSCs at passage 6 with 70–80% confluency were harvested for all experiments. Human PC3 prostate cancer cells, human smooth muscle cells (SMCs), human umbilical vein endothelial cells (HUVECs), and THP-1 human myeloid cells were purchased from ATCC and expanded following ATCC instructions. PC3 cells were transduced with the lentiviral vectors carrying firefly luciferase 2 (Luc2) and tdTomato [22] at a multiplicity of infection (MOI) of 10 virus particles per cell in the presence of 8 μg/ml polybrene, and tdTomato^+^ PC3 cells were sorted by fluorescence-activated cell sorting (FACS) (Supplementary Methods and Fig. S3) for in vivo tracing.

### Preparation and in vitro characterization of DiI-labeled nanovesicles

To prepare fluorescently labeled nanovesicles and nanoghosts for in vitro assays, iPSC-MSCs (1 × 10^6^/mL) were labeled with 5 μl/mL DiI cellular membrane labeling-solution (ThermoFisher, D-282) for 20 min at 37 °C. For nanoghost preparation, iPSC-MSCs were hypotonically treated with tris-magnesium buffer followed by mild homogenization to make ghost cells without cytosol. Following the published protocols [7, 8, 23], ghost or intact iPSC-MSCs were extruded through 10, 5, 3, 1.2, and 0.4 μm polycarbonate membranes, and nanoghosts or nanovesicles were isolated by ultra-centrifugation for 45 min at 150,000×*g* at 4 °C. The sizes of these EV-mimics were analyzed using Nanosight LM 10 Nanoparticle Tracking Analysis System (Malvern). To examine the selective uptake of nanoparticles by PCa cells in vitro, 1 × 10^10^ DiI-labeled nanoghosts, nanovesicles, or liposomes (FormuMax, F60103F-DI) were incubated with 1 × 10^5^ PC3 cells, SMCs, or HUVECs in 1 ml DMEM for 15 min, 1 h, or 3 h, then cells were washed with phosphate-buffered saline (PBS) three times and analyzed by flow cytometry for DiI signal. The selective uptake of DiI-labeled nanoparticles by PC3 cells vs. SMCs or HUVECs was quantified as the log odds ratio (LOR) as reported [8].

### Preparation and in vitro characterization of nanovesicles labeled with renilla luciferase

To trace nanovesicle content, we constructed a lentiviral vector encoding cytoplasmic renilla luciferase (rLuc) and GFP under the control of ubiquitous EF1α and PGK promoter, respectively. The rLuc cDNA was amplified from plasmid pRL Renilla Luciferase reporter vector (Promega) by PCR, linked into BamHI site of pCDH-CMV-MCS-EF1-copGFP (System Biosciences, CD511B-1) backbone, and then cloned again into the EcoRI + NotI site of plasmid backbone pCDH-EF1-MCS-BGH-PGK-GFP-T2A-Puro (System Biosciences, CD550A-1). Lentiviral vectors carrying rLuc and GFP were prepared by transfecting 293T cells (ATCC) with the above pCDH-EF1-mLuc-PGK-GFP plasmid with pPACK packaging plasmid mix and concentrated by PEG-it virus precipitation solution (System Biosciences). Human iPSC-MSCs were transduced with the rLuc-GFP lentivirus at an MOI of 10 with 8 μg/ml polybrene, and GFP^+^ cells were sorted by FACS to expand. NVs were reconstructed from non-transduced and GFP^+^ rLuc-transduced iPSC-MSCs and absorbed on 4 μm aldehyde/sulfate-latex beads (ThermoFisher) to examine GFP expression with FC500 flow cytometer (Beckman Coulter). To determine the correlation between rLuc signal intensities and rLuc-NV numbers before or after uptake by cells, rLuc-NVs were incubated alone or with 1 × 10^4^/well PC3 cells in 96-wells plate for 3 h at 37 °C, and the luminescence was measured after incubation with rLuc substrate Coelenterazine (Sigma, 2.5 μg/ml) for 15 min with a FLUOstar Omega plate reader (BMG LABTECH).

### Animals

Male NU/J athymic nude mice were purchased from the Jackson Laboratory. Subcutaneous and bone metastatic PCa mouse models were generated by injecting 1 × 10^5^ Luc2-PC3 cells subcutaneously at the right flank or 5 × 10^4^ Luc2-PC3 cells intratibially into the right hind leg of these mice at 9–12 weeks old. The animal numbers are 4 mice per group for biodistribution assays and 6 mice per group for assays on therapeutic effects as reported [7, 24]. The in vivo Luc2 signals were imaged weekly with IVIS Lumina III System (PerkinElmer) at 15 min after subcutaneous injection of D-Luciferin (150 ng/g body weight in 100 μl PBS).

### Biodistribution of nanovesicles in mice carrying PCa

Nanovesicles for biodistribution assays were labeled with either rLuc inside them or the near-infrared fluorescent dye DiR (ThermoFisher, D-12731) on their surface as reported [24] with procedures similar to DiI labeling. These nanovesicles were PEGylated as reported [8] by incubating in PBS containing Methoxy-polyethylene glycol (PEG) succinate N-hydroxysuccinimide (Sigma, 85,976) at room temperature for 2 h with gentle agitation. The PEGylation was stopped by adding 100 mg I-Lysine (Sigma), and then unreacted PEG, excess lysine and reaction by-products were eliminated by buffer exchange over a Micro-Bio Spin P-30 column (Bio-Rad) equilibrated with TM-buffer (pH 8.6, Sigma). Mice carrying established subcutaneous or bone PCa were randomly grouped and intraperitoneally (IP) injected with PEGylated DiR-NVs, rLuc-NVs or DiR-liposomes (FormuMax, F60203F-DR) at the dose of 1 × 10^10^ particles/gram (p/g) as optimized for DiR-labeled MSC EVs [24]. The numbers of NVs and liposomes were quantified by nanoparticle tracking analysis with NanoSight NS300 (Malvern). The biodistribution of DiR-labeled liposomes and nanovesicles was examined by in vivo and ex vivo imaging of DiR fluorescence with IVIS Lumina III System (PerkinElmer). For in vivo imaging of rLuc signals, ViviRen substrate (2 μg / g body weight in 200 μl PBS) was IP injected, and luminescent images were taken 15 min later with IVIS Lumina III System. The intensities of DiR or rLuc signals in tumor and non-tumor regions were quantified with the Living Image software (PerkinElmer).

### Preparation and in vitro characterization of docetaxel-nanovesicles (Dxl-NVs)

iPSC-MSCs were pre-incubated with 5 μg/mL Dxl (Sigma, PHR1883) for 24 h or not pretreated, and then broken down by serial extrusion in the presence of 50, 100, or 200 μg/mL Dxl to make Dxl-loaded NVs. The amount of Dxl loaded into NVs was determined by UV spectrometry at 230 nm as reported [25] using a spectrofluorometer (ThermoFisher). After incubation in 37 °C PBS containing 10% pooled human serum (Sigma) or 4 °C PBS for a series of periods, the supernatant was isolated by ultra-centrifugation at 100,000×*g* for 90 min at 4 °C using Sorvall WX Floor Ultra Centrifuge (Thermo) to measure Dxl release from NV-Dxl by UV spectrometry. Dxl-resistant PC3 cells were established as reported [26]. 5 × 10^3^/well parent PC3 cells, Dxl-resistant PC3 cells, or THP-1 human myeloid cells were seeded into 96-well plates, incubated with empty NVs, NV-Dxl, or free Dxl at a series of concentrations for 72 h, and then analyzed with PrestoBlue Cell Viability Assay (ThermoFisher).

### Therapeutic effects of NV-Dxl

Mouse models of subcutaneous and bone metastatic PC3 PCa were established as mentioned above. Sizes of subcutaneous tumors were measured every 4 days with calipers, and tumor volumes were calculated with the modified ellipsoid formula 1/2(length × width^2^) [27, 28]. Luc2 signals were imaged weekly and the bioluminescence intensities were quantified with the Living Image software (PerkinElmer). When subcutaneous tumors reached the size of 50 mm^3^ and Luc2 signals were clearly detectable in the injected leg by in vivo imaging, mice were randomly grouped and injected IP with PBS, free Dxl or PEGylated Dxl-NVs at the dose of 5 mg/kg body weight twice a week for 3 weeks. The blood was collected at the end point for complete blood count using the VetScan HM5 Color Hematology System (Abaxis).

### Statistical analyses

All statistical analysis and graphical generation of data were done with GraphPad Prism software. Statistical comparisons between two groups were performed with unpaired two tailed Student’s *t* tests. Comparisons involving more than two groups were performed with one-way analysis of variance (ANOVA) followed by the post hoc Bonferroni test. Comparisons involving ≥ 3 time points and ≥ 3 different treatments were performed with a repeated measures ANOVA followed by the post hoc Bonferroni test. *P* < 0.05 is considered significant. All quantified data were presented as mean ± SD.

## Results

### Nanovesicles made from iPSC-MSCs are more selectively taken up by PCa cells than nanoghosts and liposomes

**Fig. 1:**
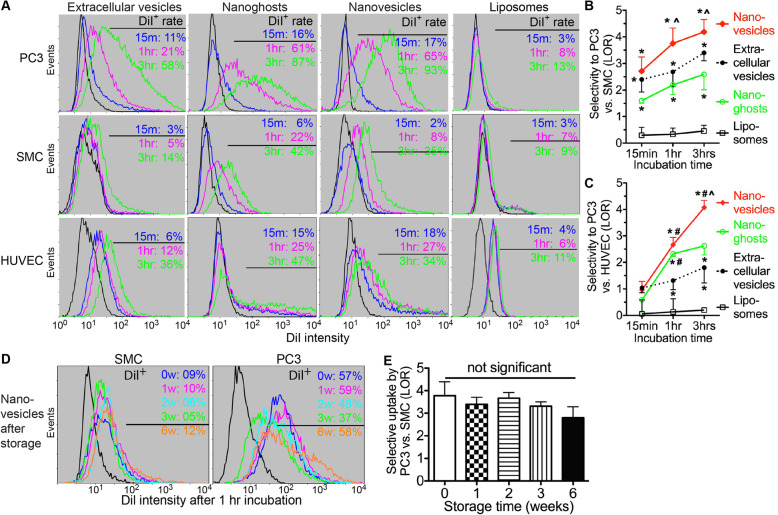
Selective uptake of iPSC-MSC nanovesicles by prostate cancer cells vs. non-tumor cells. **a** 1 × 10^5^ PC3 PCa cells, human vascular smooth muscle cells (SMCs), and human umbilical vein endothelial cells (HUVECs) in 1 ml medium were incubated with 1 × 10^10^ DiI-labeled extracellular vesicles, nanoghosts, nanovesicles, or liposomes (FormuMax) for 15 min, 1 h, or 3 h, then washed and analyzed by flow cytometry for DiI signal. **b**, **c** LORs of uptake by PC3 cells vs. SMCs or HUVECs were calculated with the formula Log2 [(% of DiI^+^ PC3/% of DiI^+^ SMC/HUVEC)/(% of DiI^−^ PC3/% of DiI^−^ SMC/HUVEC)]. **d**, **e** The selective uptake of nanovesicles stored for up to 6 weeks by PC3 cells vs. SMCs was analyzed as in **a** and **b**. *N* = 3 in all assays, *p < 0.05 vs. liposomes, ^*p* < 0.05 vs. nanoghosts, ^#^*p* < 0.05 vs. extracellular vesicles

We prepared extracellular vesicles (EVs), nanoghosts (NGs), and nanovesicles (NVs) from our iPSC-MSCs cultured under same conditions as reported [7, 8, 18, 21]. The mean size of nanovesicles is much smaller and more uniform than nanoghosts and EVs (Fig. S1A), suggesting that nanovesicles might achieve better tumor penetration due to smaller particle size [29]. The yield of nanovesicles is about 3-fold of that of nanoghosts and 6-fold of EVs in term of particle numbers (Fig. S1B). Both nanoghosts and nanovesicles express EV surface markers ALIX and TSG101 at levels comparable to EVs (Fig. S1C-D). To trace iPSC-MSC EVs, nanoghosts, and nanovesicles in vitro, we labeled them with a lipophilic fluorescent dye DiI [8]. The DiI labeling efficiency was comparable between EVs, nanoghosts, nanovesicles, and commercially available DiI-liposomes, and the DiI signal intensities were in proportion to numbers of these particles [18]. To determine the uptake of these particles by prostate cancer cells vs. non-tumor cells, we incubated DiI-labeled and non-PEGylated liposomes, EVs, nanoghosts, or nanovesicles with human PC3 PCa cells, human smooth muscle cells (SMCs), or human umbilical vein endothelial cells (HUVECs) for 15 min, 1 h, or 3 h. After washing, percentages of DiI^+^ cells were determined by flow cytometry, and the selective uptake by PC3 cells was quantified as log odds ratios (LOR) vs. SMCs or HUVECs as reported [8]. The uptake of liposomes by PC3 cells was comparable to non-tumor cells as expected, whereas iPSC-MSC EVs, nanovesicles, and nanoghosts were selectively taken up by PC3 cells vs. either SMCs or HUVECs after various incubation periods (Fig. 1a–c). Despite the same cell source, the PC3-selective uptake of nanovesicles (LOR) is significantly higher than that of EVs and nanoghosts after incubation for 1 or 3 h, which is related to the higher uptake by PC3 cells of nanovesicles than EVs and the lower uptake by non-tumor cells of nanovesicles than nanoghosts (Fig. 1a–c). The selective uptake of nanovesicles by PC3 cells vs. SMCs was validated by confocal microscope imaging of GFP-transduced cells incubated with DiI-labeled nanovesicles for 1 or 3 h (Fig. S2). We have confirmed that iPSC-MSC nanovesicles maintain their size, charge, and selective uptake by breast cancer cells after prolonged storage [18]. Consistently, after storage at 4 °C for 1, 2, or 3 weeks or at − 80 °C for 6 weeks, the selective uptake of iPSC-MSC nanovesicles by PC3 cells vs. SMCs was not significantly decreased compared to fresh nanovesicles resuspended in PBS at 4 °C overnight (Fig. 1d, e). These data indicate that iPSC-MSC nanovesicles are superior to EVs and nanoghosts for PCa-targeting and maintain PCa-targeting capacity after storage; therefore we focus on iPSC-MSC nanovesicles for all following experiments.

### Systemically infused iPSC-MSC nanovesicles accumulate in subcutaneous PCa with higher efficacy than liposomes

**Fig. 2:**
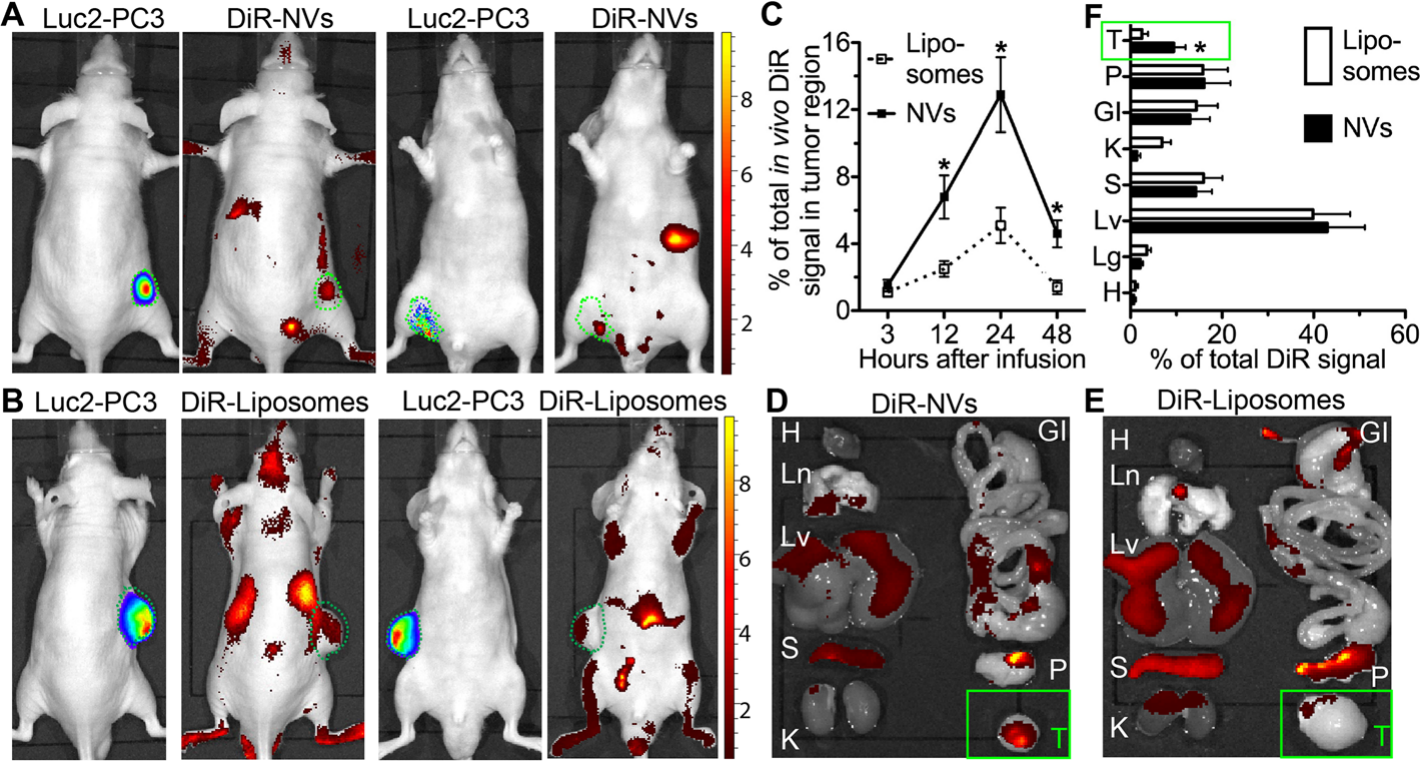
Biodistribution of systemically infused DiR-NVs in mice carrying subcutaneous prostate cancer. Nude mice carrying subcutaneous Luc2-PC3 tumors were randomly grouped and IP infused with PEGylated liposomes or NVs labeled with DiR. **a**, **b** The representative in vivo imaging of Luc2 and DiR signals at 24 h after infusion from both the dorsal and ventral sides. **c** The relative intensities of in vivo DiR signals in Luc2^+^ tumor region and non-tumor regions were quantified at 3, 12, 24, and 48 h after infusion as percentages of total DiR signals. **d**–**f** Tumors (T) and organs were collected 24 h after infusion for ex vivo imaging and consequent quantification. H, heart; Ln, lungs; Lv, liver; S, spleen; K, kidneys; GI, gastrointestinal tract; P, pancreas. *N* = 4 in all assays, *p < 0.05

For in vivo biodistribution assays, nanovesicles were firstly labeled with a near-infrared lipophilic fluorescent dye DiR as reported for EVs [24], PEGylated as reported for BM-MSC nanoghosts [8], and compared with DiR-labeled liposomes (FormuMax) PEGylated in the same way as FDA-approved liposomes to deliver a chemotherapy drug (Doxil, 5% PEG 2000-DSPE) [30]. Subcutaneous xenograft models are widely used for PCa study to facilitate the monitoring of PCa growth and the harvest of PCa tumors. Nude mice carrying subcutaneous Luc2-PC3 PCa were randomly grouped and injected intraperitoneally (IP) with DiR-labeled liposomes or nanovesicles. In vivo Luc2 and DiR imaging indicated that DiR signals in Luc2^+^ tumor region were significantly higher in the NV group 12, 24, and 48 h after infusion than in the liposome group, while strong DiR signals in both groups were also present in the upper abdomen area containing the mononuclear phagocytic system (MPS) organs such as the liver and spleen (Fig. 2a–c). Tumors and major organs were harvested 24 h after infusion for ex vivo DiR imaging. The tumor DiR signals were significantly higher in the NV group than in the liposome group, whereas DiR signals in the liver and spleen are comparable high in both groups (Fig. 2d–f).

**Fig. 3:**
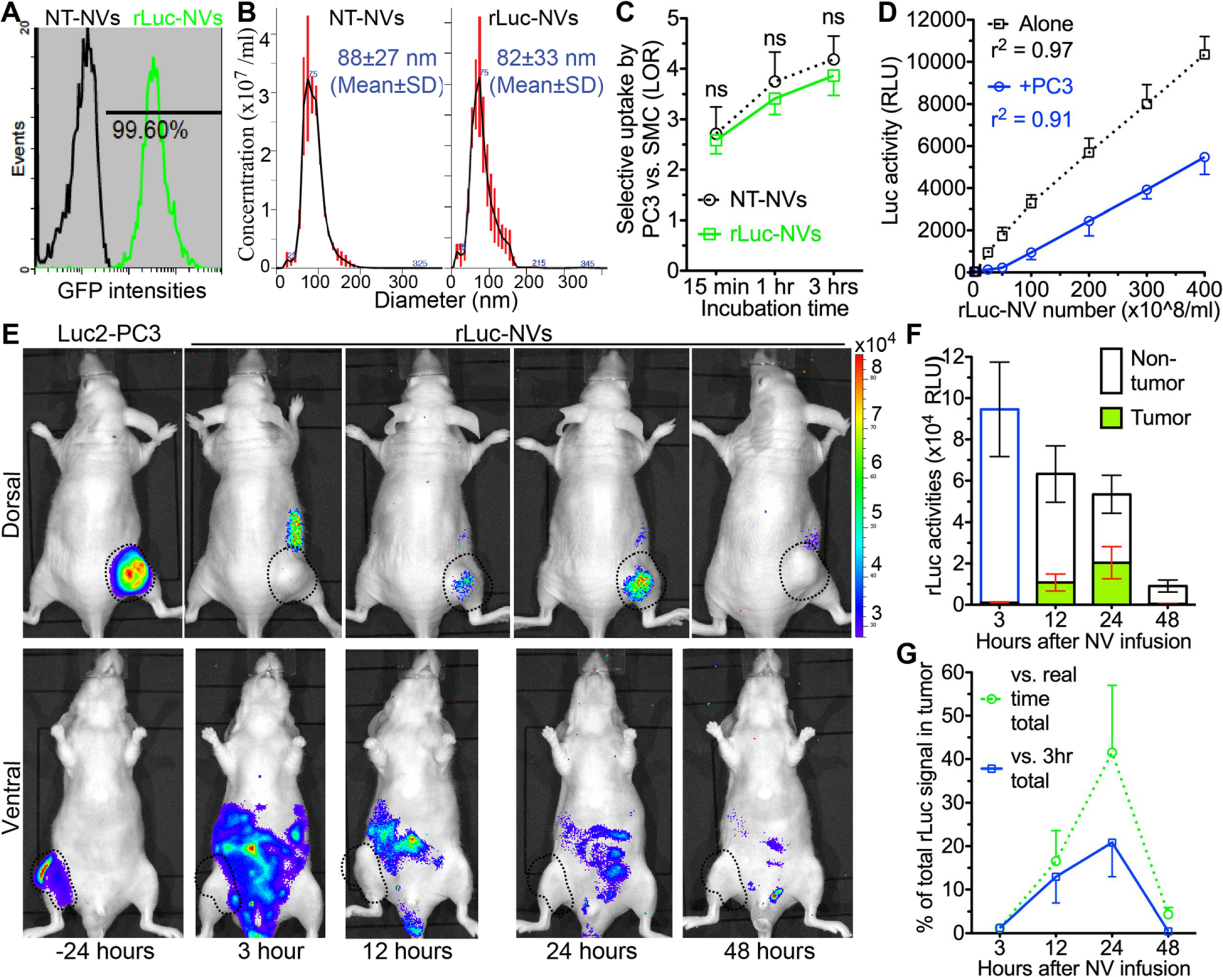
Biodistribution of systemically infused rLuc-NVs in mice carrying subcutaneous prostate cancer. **a** The expression of GFP in NVs made from rLuc-GFP-transduced and non-transduced (NT) iPSC-MSCs was examined with flow cytometry. **b** The size distribution of NVs made from rLuc-transduced and NT iPSC-MSCs was determined with Nanosight nanoparticle tracking system. **c** The selective uptake of NT-NVs and rLuc-NVs by PC3 cells vs. SMCs (LOR) was measured as in Fig. 1. **d** In vitro bioluminescence activities of rLuc-NVs without or with pre-incubation with PC3 cells for 3 h were measured at 15 min after addition of rLuc substrate. RLU, relative luminescence units. **e** In vivo imaging of rLuc-NVs at 3, 12, 24, and 48 h after IP infusion of rLuc-NVs from ventral and dorsal side. **f** Absolute intensities of rLuc signals in Luc2^+^ tumor and non-tumor regions. **g** The percentages of tumor rLuc signals vs. total rLuc signals at corresponding time points (green dashed line) and vs. total rLuc signals at 3 h (blue solid line). *N* = 4 in all assays; ns, not significant

Since assays on DiR only reflect the distribution of membrane components of nanovesicles, we then labeled contents of nanovesicles with cytoplasmic renilla luciferase (rLuc) that can be clearly distinguished from Luc2 signals in PC3 cells based on distinct substrates and bioluminescent properties. Nanovesicles were reconstructed form iPSC-MSCs transduced with a lentiviral vector encoding cytoplasmic rLuc and green fluorescent protein (GFP), and almost all rLuc-labeled NVs express GFP as indicated by flow cytometry assay (Fig. 3a). These rLuc-nanovesicles were comparable to non-transduced (NT) nanovesicles in size and selective uptake by PC3 cells vs. non-tumor cells (Fig. 3b, c). We then examined the luciferase activity of rLuc-labeled NVs in vitro with or without co-culture with PC3 cells. Under both conditions, the intensities of rLuc signals were correlated to nanovesicle numbers in linear relationships (Fig. 3d, *r*^2^ > 0.91). These rLuc-nanovesicles were PEGylated and IP injected into mice carrying subcutaneous PC3 tumors. The in vivo imaging indicated that at 12 and 24 h after NV infusion, strong and localized rLuc signals were present at Luc2^+^ tumor regions (Fig. 3e–g). Compared with tumor DiR signals, the relative intensity of tumor rLuc signals was higher at 12 and 24 h after NV infusion but decreased faster. Similar to DiR signals, strong and dispersed rLuc signals were also present in the abdomen area containing MPS organs (Fig. 3e–g). The DiR and rLuc imaging data together indicated the selective accumulation of infused nanovesicles in subcutaneous PCa and MPS organs.

### Systemically infused iPSC-MSC nanovesicles accumulate in bone metastatic PCa with higher efficacy than liposomes

**Fig. 4:**
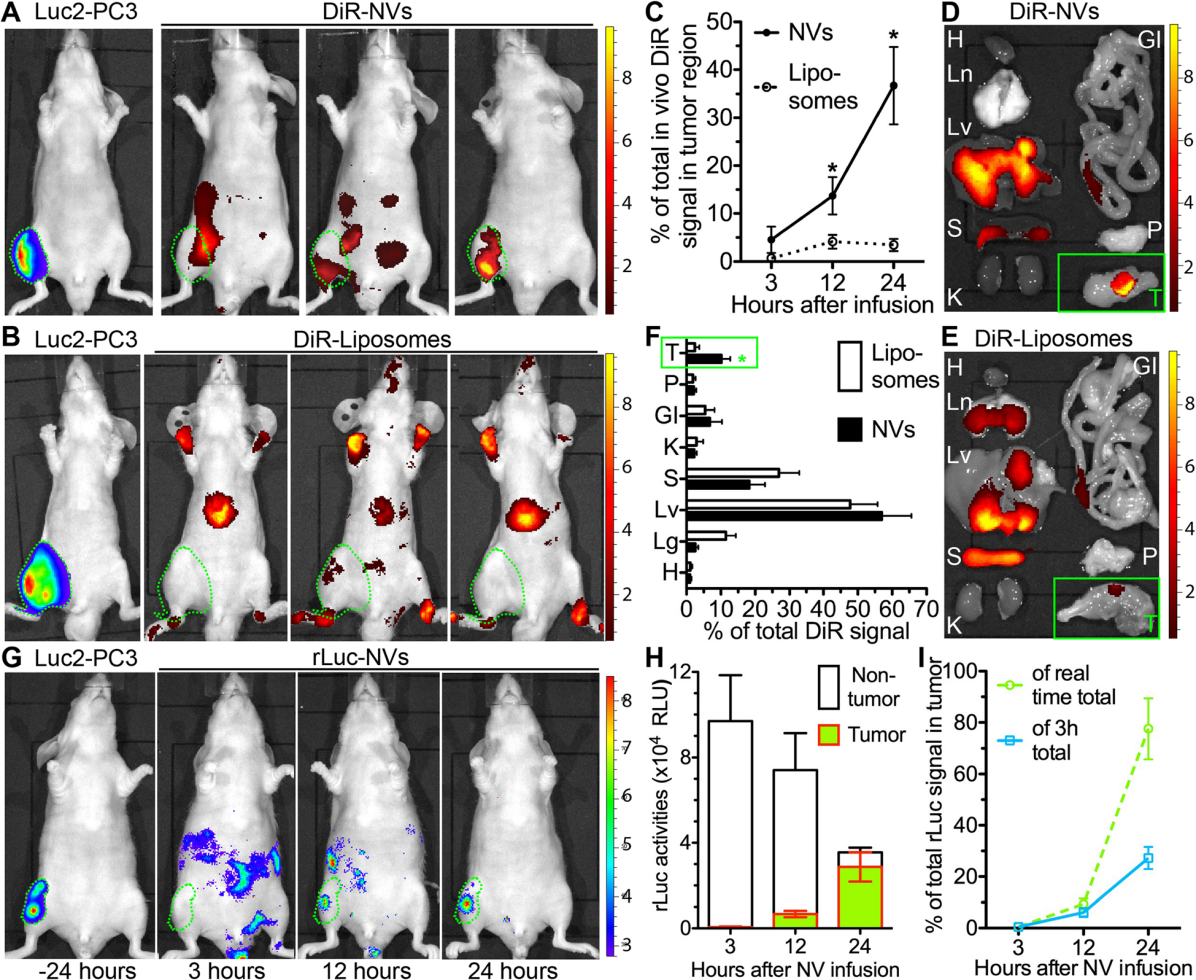
Biodistribution of systemically infused NVs in mice carrying bone metastatic PC3 cancer. Nude mice carrying Luc2-PC3 tumor in the right hind leg were IP infused with 1 × 10^10^ p/g DiR-liposomes or DiR-NVs (**a**–**f**), or rLuc-NVs (**g**–**i**). **a**, **b** The representative in vivo imaging of Luc2 and DiR signals at 3, 12, and 24 h after infusion. **c** The relative intensities of in vivo DiR signals in Luc2^+^ tumor region and non-tumor regions were quantified as percentages of total DiR intensities at 3, 12, 24, and 48 h after infusion. **d**–**f** Tumor-carrying legs (T) and organs were collected 12 h after infusion of DiR-liposomes or DiR-NVs for ex vivo imaging and consequent quantification. H, heart; Ln, lungs; Lv, liver; S, spleen; K, kidneys; GI, gastrointestinal tract; P, pancreas. **g** In vivo imaging of rLuc signals at 3, 12, and 24 h after IP infusion of rLuc-NVs. **h** Absolute intensities of rLuc signals in tumor and non-tumor regions. **i** The percentages of tumor rLuc signals vs. total rLuc signals at corresponding time points (green dashed line) and vs. total rLuc signals at 3 h (blue solid line). *N* = 4, *p < 0.05 vs. the liposome group

The bone metastatic PCa mouse model was generated by intratibial injection of Luc2-PC3 cells into nude mice, and then DiR-labeled NVs or liposomes or rLuc-labeled NVs were injected IP into randomly grouped mice carrying bone metastatic PCa. In vivo DiR imaging indicated that DiR signals in Luc2^+^ bone PCa regions were much stronger in the NV group than in the liposome group at 12 and 24 h after infusion, while strong DiR signals in both groups were also present in the upper abdomen area containing the MPS organs (Fig. 4a–c). The ex vivo DiR imaging of tissues harvested 12 h after the infusion confirmed much higher relative DiR signals within the PCa-carrying leg in the NV group than in the liposome group, and also revealed high DiR signals in the liver and spleen comparable between these two groups (Fig. 4d–f). The rLuc imaging confirmed that IP injected rLuc-NVs accumulated in bone metastases despite a much faster decrease of rLuc signals than DiR signals (Fig. 4g–i). These data indicated that systemically injected iPSC-MSC NVs are capable of targeting metastatic PCa with a selectivity superior to liposomes. However, similar to all other nanoparticles including bone marrow MSC EVs [24] and nanoghosts [8, 9], a large portion of the infused NVs is taken up by MPS organs despite the routine PEGylation to increase their stealth, which need be decreased for future applications.

### Nanovesicles efficiently encapsulated docetaxel and enhanced its cytotoxic effects on resistant prostate cancer cells

**Fig. 5:**
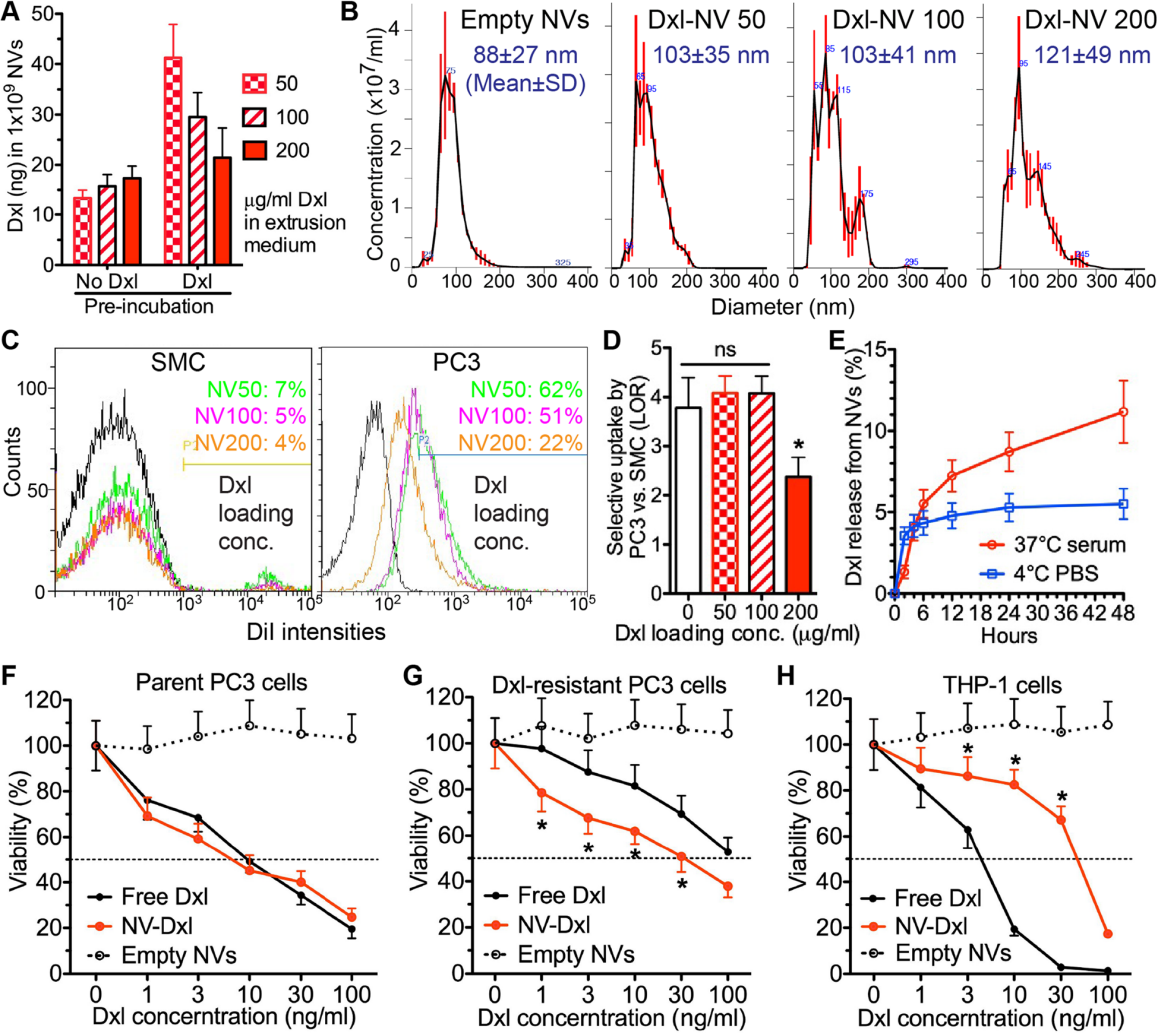
The optimization and in vitro characterization of docetaxel-loaded nanovesicles. **a** iPSC-MSCs were not pretreated or pretreated with 5 μg/mL Dxl for 24 h, and then broken down by serial extrusion in 50, 100, or 200 μg/mL Dxl to make Dxl-NVs. Dxl-NVs were isolated by ultra-centrifugation and the Dxl loading in NVs was measured by UV spectrometry. **b** Sizes of empty NVs or Dxl-NVs extruded in 50, 100, or 200 μg/mL Dxl (NV 50, NV 100, NV 200) were determined by Nanosight nanoparticle tracking analyses. **c**, **d** Effects of Dxl loading on selective uptake of DiI-labeled NVs by PC3 cells vs. SMCs were examined by flow cytometry and the LORs were calculated as in Fig. 1. **e** NV-Dxl was incubated in 37 °C PBS containing 10% human serum or 4 °C PBS for a series of periods, and the supernatant was isolated by ultra-centrifugation to measure Dxl release by UV spectrometry. Parent PC3 cells (**f**), Dxl-resistant PC3 cells (**g**), or THP-1 human myeloid cells (**h**) were incubated with empty NVs, NV-Dxl, or free Dxl at a serial of concentrations for 72 h and then analyzed with PrestoBlue Cell Viability Assay (ThermoFisher). *N* = 3 in all assays, *p < 0.05 vs. all other groups

Docetaxel (Dxl) is a first-line drug for metastatic prostate cancer [31], but its dose is limited by toxicities to non-tumor cells such as myeloid cells [32]. Furthermore, Dxl resistance is common in advanced PCa patients [33]. Based on the protocol for loading Dxl into EVs from tissue-derived MSCs [34], iPSC-MSCs were pretreated with 5 μg/mL Dxl for 24 h or not pretreated; and then broken down into NVs in solutions containing 50, 100, or 200 μg/ml Dxl as reported for loading a similar drug, paclitaxel, into EV mimics [35]. The maximal Dxl loading into NVs was achieved by extrusion of Dxl-pretreated iPSC-MSCs in 50 μg/mL Dxl (Fig. 5a). In comparison to empty NVs, Dxl loading at 50 or 100 μg/ml did not significantly affect the size distribution of NVs (Fig. 5b) and the selective uptake of NVs by PC3 cells vs. SMCs (Fig. 5c, d), whereas Dxl loading at 200 μg/ml slightly increased the NV size and significantly decreased the selective uptake of NVs by PC3 cells. Therefore, we chose NV-Dxl made by extrusion of Dxl-pretreated iPSC-MSCs in 50 μg/mL Dxl for all following experiments. The release kinetics of Dxl from NV-Dxl indicated that in both 4 °C PBS and 37 °C serum most Dxl remained inside NVs for up to 48 h (Fig. 5e). In parent PC3 cells, the cytotoxicity of NV-Dxl was comparable to free Dxl, whereas empty NVs displayed no cytotoxicity at amounts equal to those of NV-Dxl (Fig. 5f). In Dxl-resistant PC3 cells established as reported [26], the cytotoxicity of NV-Dxl was significantly stronger than free Dxl: the half maximal inhibitory concentration (IC50) of free Dxl is higher than 100 ng/ml, whereas that of NV-Dxl is around 30 ng/ml (Fig. 5g). Dxl frequently causes toxicities to non-tumor cells such as myeloid cells, and the consequent severe neutropenia is a major dose-limiting adverse effect of Dxl [32]. Therefore, we examined the toxicity of NV-Dxl and free Dxl on human THP-1 myeloid cells. As indicated by the viability assay, NV encapsulation significantly decreased Dxl toxicity on THP-1 cells at concentrations between 3 to 30 ng/ml (Fig. 5h). These data suggest that NV-Dxl could efficiently overcome Dxl-resistance of PCa in vivo at tolerable doses.

### Therapeutic and adverse effects of nanovesicles loaded with docetaxel in the subcutaneous and bone metastatic PC3 PCa mouse models

**Fig. 6:**
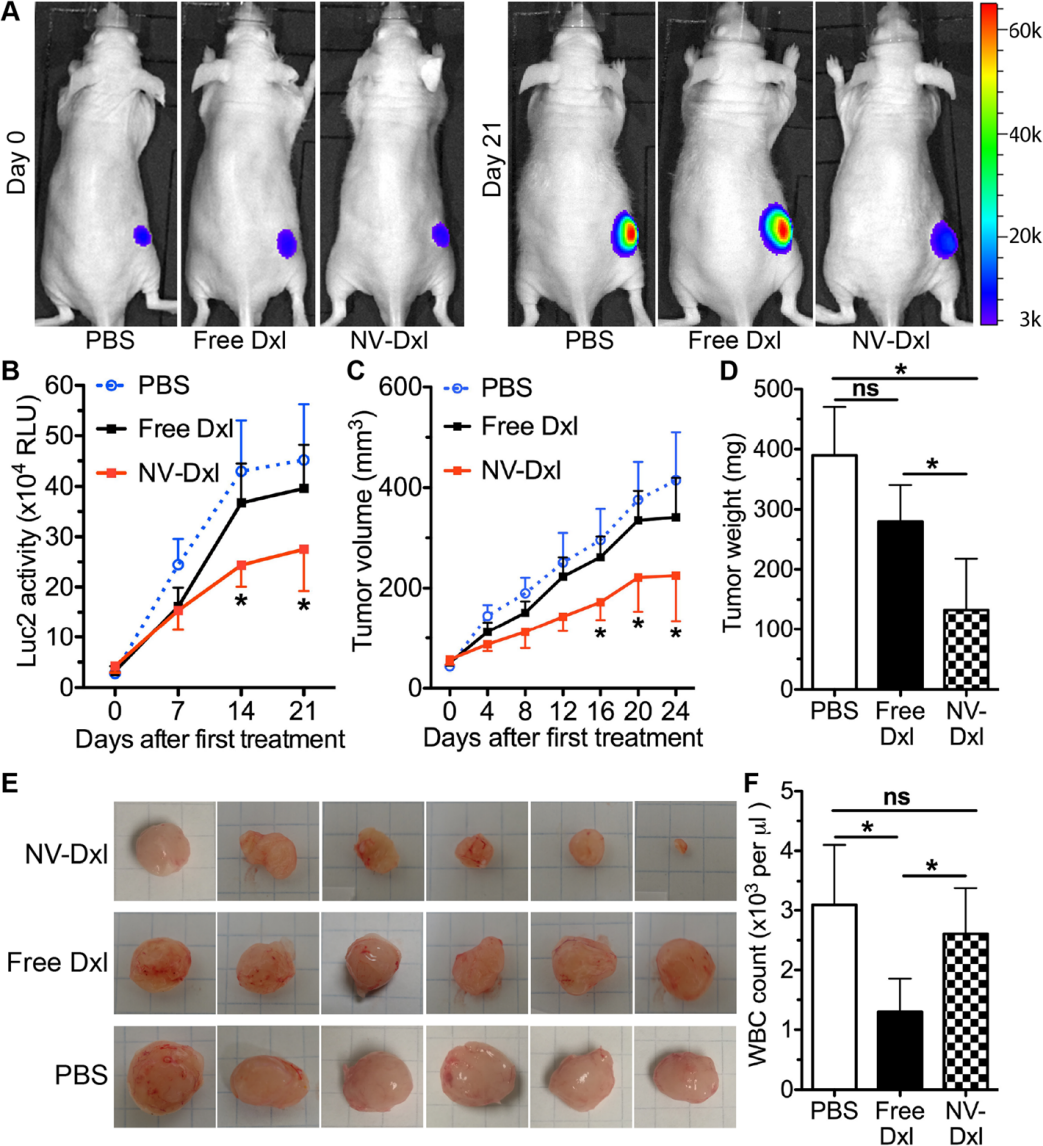
Therapeutic effects of NV-Dxl in the mouse model of subcutaneous prostate cancer. **a** The representative in vivo Luc2 bioluminescence images before and by the end of treatments. **b** The quantification of weekly in vivo Luc2 bioluminescence intensities during treatment. **c** Tumor sizes were measured every 4 days till the end point of 24 days after treatment. **d**, **e** Tumors were harvested and weighed at the end point. **f** The blood was collected at the end point to count white blood cell (WBC). *N* = 6, *p < 0.05 vs. the free Dxl group and the PBS group; ns, not significant

Both PCa mouse models were generated as mentioned above with Luc2-PC3 cells. When subcutaneous tumors reached the size of 50 mm^3^ and Luc2 signals were clearly detectable by in vivo imaging, we IP injected PBS, free Dxl or PEGylated Dxl-NVs into randomly grouped mice at the dose of 5 mg/kg body weight twice a week for 3 weeks. The dose of NV-Dxl was chosen as reported for Dxl carried by synthesized nanoparticles that effectively inhibited the progression of subcutaneous PCa and reduced hematological toxicity in a mouse model [36]. The tumor sizes were monitored every 4 days and Luc2 signals were imaged weekly. Compared with free Dxl and PBS treatment, NV-Dxl significantly decreased the Luc2 signal intensity and tumor volume after 2 weeks of treatment (Fig. 6a–c) and reduced the tumor weight at the end point of 24 days after treatment (Fig. 6d, e). Consistently, tumors from mice treated with NV-Dxl contain more TUNEL-positive cells than in free Dxl or PBS group, indicating an increase in apoptosis (Fig. S5). Meanwhile, the white blood cell (WBC) count at the end point was significantly decreased by free Dxl but not by NV-Dxl compared with the PBS group (Fig. 6f).

**Fig. 7:**
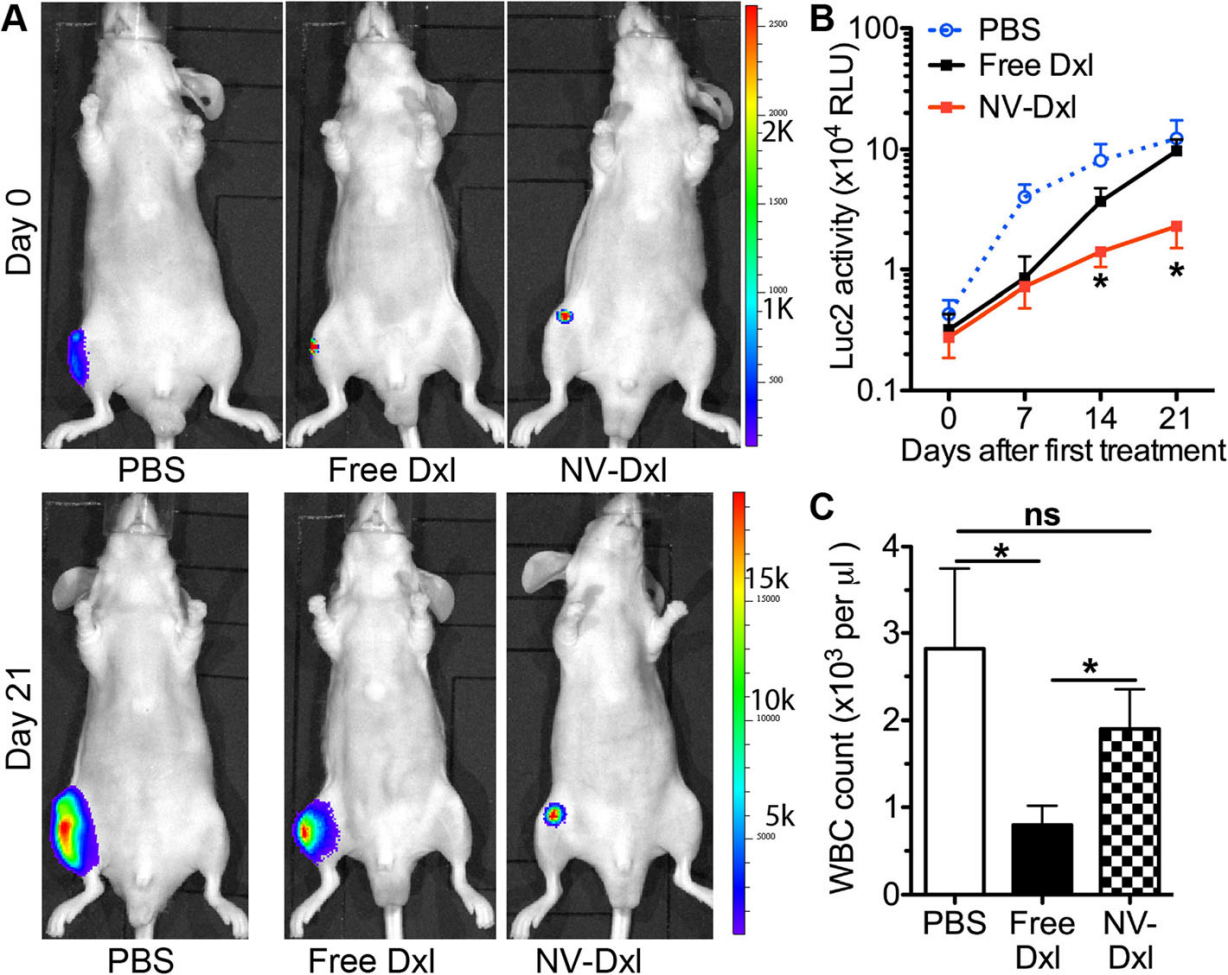
Therapeutic effects of NV-Dxl in the mouse model of bone metastatic prostate cancer. **a** The representative in vivo Luc2 bioluminescence images before and by the end of treatments. **b** The quantification of weekly in vivo Luc2 bioluminescence intensities during treatments. **c** The blood was collected at the end point to count white blood cell (WBC). *N* = 6, *p < 0.05 vs. the free Dxl group and PBS group; ns, not significant

The establishment and progression of bone metastatic PCa was monitored by in vivo imaging of Luc2 bioluminescence weekly. When Luc2 signals were clearly detectable at the leg injected with Luc2-PC3 cells, mice were randomly grouped and intraperitoneally injected with PBS, free Dxl, or PEGylated Dxl-NVs at a dose of 5 mg/kg body weight twice a week for 3 weeks. Compared with free Dxl and PBS treatment, NV-Dxl significantly decreased the Luc2 bioluminescence intensity after 2 weeks of treatment (Fig. 7a, b). Meanwhile, the white blood cell count at the end point of 21 days after treatment was significantly decreased by free Dxl but not by NV-Dxl compared with the PBS group (Fig. 7c). These data suggest that NV encapsulation can increase therapeutic effects of Dxl on metastatic PCa and decrease toxic effects of Dxl on white blood cells.

## Discussion

We reported recently that iPSC-MSCs circumvent donor variations, expansion limitations, and the decreased expression of cancer-targeting surface molecules during expansion in tissue-derived MSCs; therefore, iPSC-MSCs are a reliable source of cancer-targeting EV-mimics [18]. Moreover, nanovesicles made from intact iPSC-MSCs have a much higher production yield and smaller and more consistent sizes compared with EVs and nanoghosts made from membrane-only ghost cells [18]. Here, we report that iPSC-MSC nanovesicles are more selectively taken up by prostate cancer (PCa) cells than EVs, nanoghosts, and liposomes, the mainstream drug carrier for cancer nanomedicine. Moreover, nanovesicles can effectively deliver cytoplasmic components of iPSC-MSCs into PCa as indicated by the rLuc tracing assays. These advantages make nanovesicles a better choice than EVs and nanoghosts for targeting prostate cancer.

In both subcutaneous and bone metastatic PCa mouse models, human iPSC-MSC nanovesicles selectively accumulate in PCa with much higher efficiencies than liposomes. The PCa-targeting capacity of MSCs and MSC EV mimics is related to multiple surface proteins that interact with molecules abundant in PCa tumors [8, 9]. Advanced PCa cells express high levels of Integrin α3, α6, and β1 (ITGA3/A6/B1) [37, 38], EphA2 [39, 40], and connexins (Cx) such as Cx43/45 [41] at their surface, and also increase levels of extracellular matrix (ECM) components including fibronectin (FN) [42], osteopontin (OPN) [43], and hyaluronan [44] in tumor stroma. These over-expressed molecules contribute to the maintenance of cancer stem cells, metastasis, and poor clinical outcome. MSCs and their EVs or EV mimics carry multiple ligands of above molecules including Integrin α4, α11 and β1 (ITGA4/A11/B1) [8, 9], CD44, CD63, TSPAN4, ICAM1, VCAM1, CD9, CD81, and Cx43 [18] (Fig. S4). We have confirmed the high levels of these ligands in our iPSC-MSCs and derived EV mimics [18]. Moreover, the common PTEN-deficiency in metastatic PCa cells including PC3 cells greatly promotes macropinocytosis [45], which in turn dramatically enhances uptake of adjacent EVs and EV-mimics [5]. These two mechanisms together make iPSC-MSC nanovesicles a promising carrier for the targeted delivery of anti-cancer agents into metastatic PCa.

However, like EVs and all other nanoparticles, a large portion of nanovesicles accumulate in the mononuclear phagocytic system (MPS) organs despite the routine PEGylation. The PEG layer can reduce the binding of synthetic nanoparticles to phagocytes and prolong their circulation time and also inhibits active cancer targeting and cellular uptake mediated by targeting ligands and consequent intracellular delivery of payloads [46]. Since PCa express much higher levels of matrix metalloproteinases (MMP) than non-tumor tissues [47], the shielding of nanovesicles with MMP-sensitive PEG [48–50] is expected to lead to the cancer-specific degradation of PEG, which can enhance the uptake of nanovesicles by PCa cells while inhibiting that by MPS cells. Another promising approach is pretreating MSCs with proinflammatory cytokines before extrusion: in the mouse model of subcutaneous PC3 PCa, pretreatment of BM-MSCs with TNFα and IL1β significantly increased the accumulation of MSC nanoghosts in PCa but not in MPS organs, which is likely related to slight changes in expression levels of multiple membrane proteins [51].

The current guideline of International Society of Geriatric Oncology recommends docetaxel (Dxl) as the standard of care for metastatic PCa [31], but some patients cannot tolerate the toxicity of Dxl and all PCa patients will ultimately develop resistance to Dxl [33]. The encapsulation with nanovesicles significantly improved the cytotoxicity of Dxl on cultured Dxl-resistant PCa cells, which is likely through diverting enhanced drug efflux mediated by over-expressed or hyperactive membrane drug transporters [5]. In both subcutaneous and bone metastatic PCa mouse models, NV-Dxl significantly inhibited the tumor growth compared with the equal dose of free Dxl and resulted in much weaker toxicity on white blood cells. The increased therapeutic efficacy of NV-Dxl is likely related to both the enhanced delivery of Dxl into PCa tumors and the decreased Dxl efflux by membrane drug transporters in PCa cells.

## Conclusion

Our data indicate that EV-mimetic nanovesicles made from iPSC-MSCs with a theoretically limitless expandability and consistent biological properties are a promising platform for targeted delivery of anti-cancer agents to improve treatment of metastatic prostate cancer.

## Supplementary Information

**Additional file 1.**

**Additional file 2.**

## Data Availability

All data generated and/or analyzed during this study are included in this published article. The iPSC-MSCs and rLuc lentiviral vector will be distributed via Material Transfer Agreements (MTAs) generated and monitored by the Texas A&M Office of Technology Commercialization.

## Abbreviations

PCa: Prostate cancer
iPSCs: Induced pluripotent stem cells
MSCs: Mesenchymal stem cells
EVs: Extracellular vesicles
NVs: Nanovesicles
SMCs: Smooth muscle cells
HUVECs: Human umbilical vein endothelial cells
PBS: Phosphate-buffered saline
LOR: Log odds ratios
PEG: Polyethylene glycol
PEGylation: A technique of covalent or noncovalent attachment of PEG to nanoparticles
FACS: Fluorescence-activated cell sorting
Dxl: Docetaxel
BM: Bone marrow
MPS: Mononuclear phagocytic system
Luc2: Firefly luciferase 2
rLuc: Renilla luciferase
RLU: Relative luminescence units
GFP: Green fluorescent protein
NT: Non-transduced
IP: Intraperitoneally
p/g: Particles/gram body weight
IC50: The half maximal inhibitory concentration
ANOVA: Analysis of variance
WBC: White blood cell
MMP: Matrix metalloproteinase
