# Supplementary material for: Extracellular vesicle mimics made from iPS cell-derived mesenchymal stem cells improve the treatment of metastatic prostate cancer
Source: Stem Cell Res Ther. 2021 Jan 7;12:29. doi: 10.1186/s13287-020-02097-5 (PMC7792192; doi:10.1186/s13287-020-02097-5)
Supplement: Supplementary file 1 — Additional file 1. [file 13287_2020_2097_MOESM1_ESM.pdf]

SUPPLEMENTARY METHODS

Fluorescence-activated cell sorting (FACS)

After transducing PC3 cells with Luc2-tdTomato and iPSC-MSCs with rLuc-GFP, $tdTomato^{+}$ and GFP^{+} cells were sorted respectively with a MoFlo Astrios High-Speed Cell Sorter (Beckman Coulter). These cells were digested into single cells with 0.25% trypsin/EDTA and resuspended in their culture media to $1x10^{6}$ cells/mL. The FACS setting are: Sort setup l00 micron, Precision 4-Way Purity, Frequency 30, Yield Mask 0, Amplitude 11.3, Purity Mask 32, Phase 0, Phase Mask 0, Drop Delay 26.91, Single cell Off, Attenuation Off, Plates Voltage 2500, Sweet Spot On, Voltage Centering 216, First Drop 235, Sheath Pressure 20, Target Gap 10. Side Stream Voltage (%): Far Left 88, Left 27, Right 34, Far Right 77. Neighboring Drop Charge (%): 2nd 9, 3rd 5, 4th 3.

Histological and immunofluorescent analysis of harvested tumors

Subcutaneous prostate cancer harvested from mice treated with PBS, free Dxl, or NV-Dxl were sliced into 10 μm thick sections. Tumor sections were stained with hematoxylin-eosin (H&E) or subjected to TUNEL (terminal deoxynucleotidyl transferase dUTP nick end labeling) assay using a fluorescein in situ cell death detection kit (Roche Cat. No. 11684795910). Sections were counterstained with DAPI (Sigma), dehydrated in ethanol, and mounted with glass cover-slips. Apoptosis was quantified by determining the percentage of positively stained cells (stained cells divided by total nuclei) in 9 randomly chosen fields per group at 200x magnification with NIS Elements image analysis software (NIKON).

SUPPLEMENTARY FIGURES

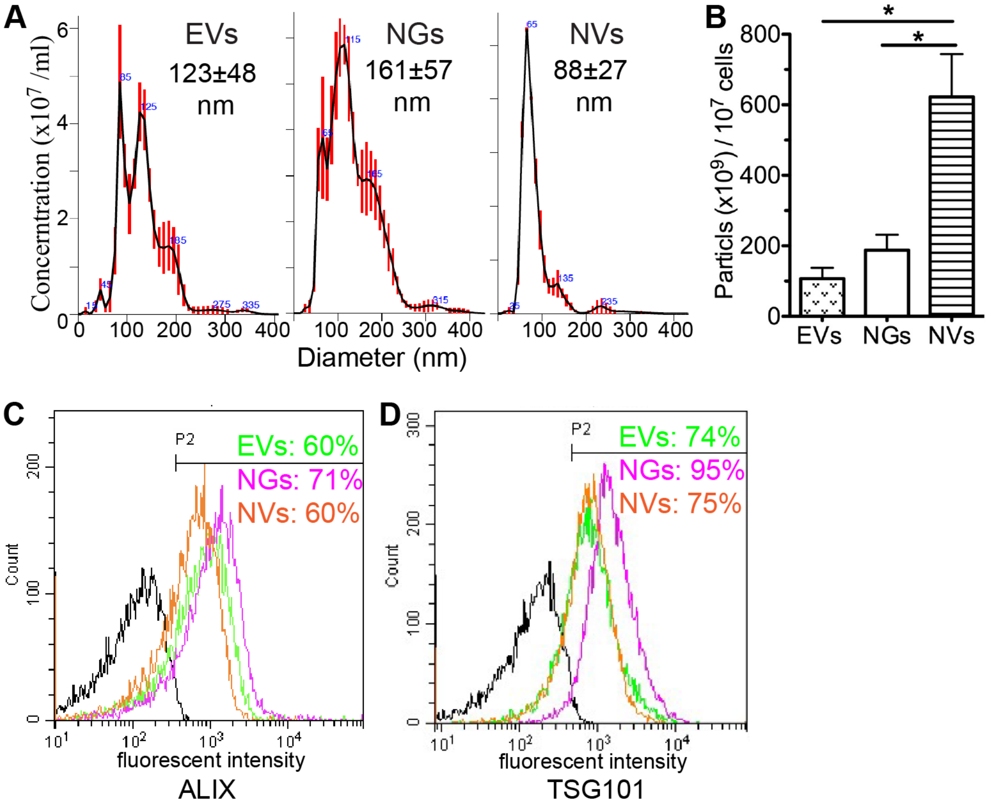

Figure S1. The characterization of iPSC-MSC EVs, nanoghosts and nanovesicles.

A-B: The sizes and production yields of iPSC-MSC EVs, nanoghosts and nanovesicles were measured with Nanosight nanoparticle tracking system. N = 3.

C-D: Flow cytometry analyses on the expression of EV markers ALIX and TSG101 on iPSC-MSC EVs, nanoghosts and nanovesicles were performed after absorption on 4 µm aldehyde/sulphate-latex beads. The black curves are the results of isotype control antibodies incubated with beads absorbed with nanoghosts.

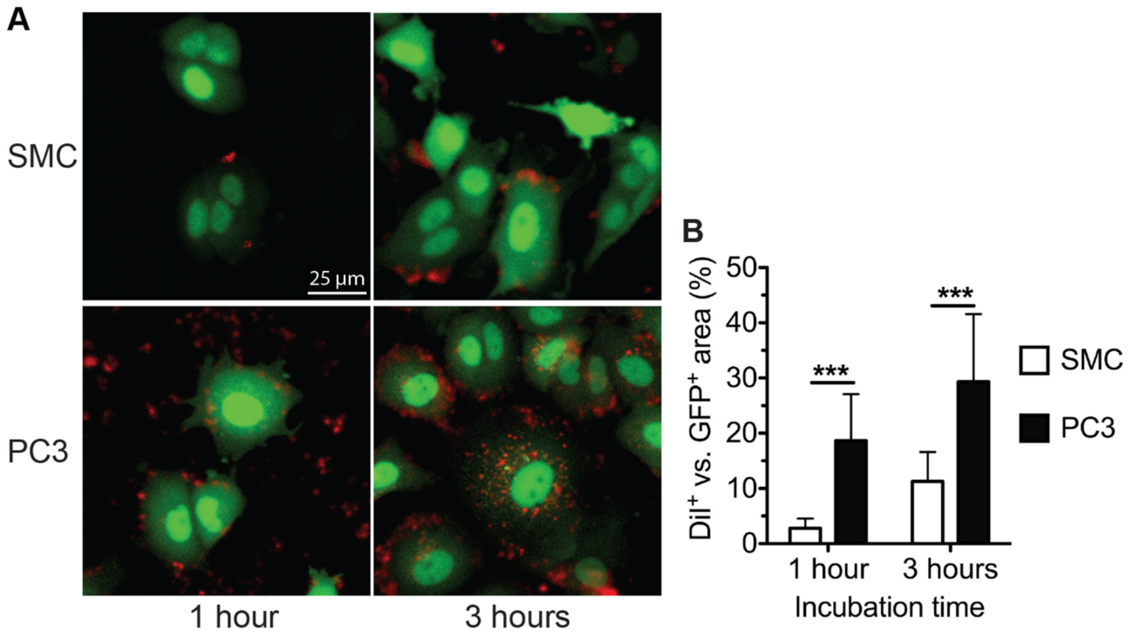

Figure S2. The confocal microscopy validated the selective uptake of iPSC-MSC nanovesicles by PC3 prostate cancer cells.

(A) PC3 cells and SMCs (human smooth muscle cells) were transduced with lentiviral vectors carrying EF1α-copGFP (System Biosciences, CD511B-1), incubated with $1x10^{10}$ /ml DiI-labeled nanovesicles for 1 or 3 hours, washed 3 times with PBS, and then imaged with confocal microscope.

(B) The percentages of DiI^+ regions in GFP^+ areas were quantified from 9 100x fields of 3 independent samples for each type of cells and each time point using Image J software. ***: $p < 0.001$.

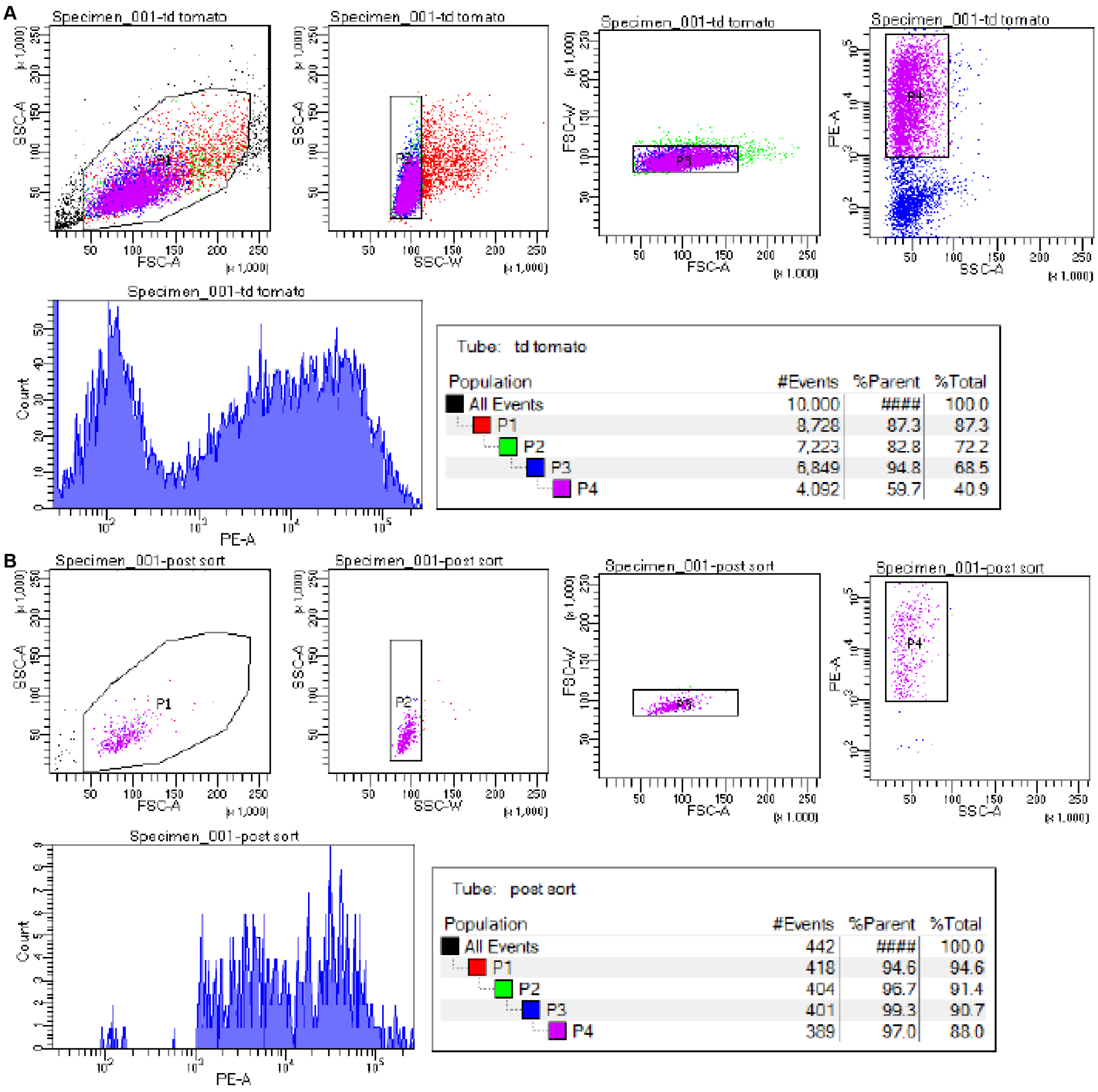

Figure S3. FACS sorting of PC3 cells transduced with Luc2-tdTomato.

A: Pre-sorting analysis. B: Post-sorting analysis.

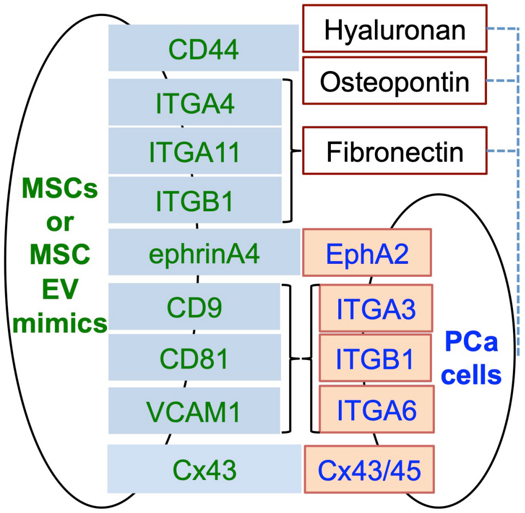

Figure S4. Scheme of surface molecules related to the targeting of MSCs and MSC EVs or EV mimics to metastatic PCa.

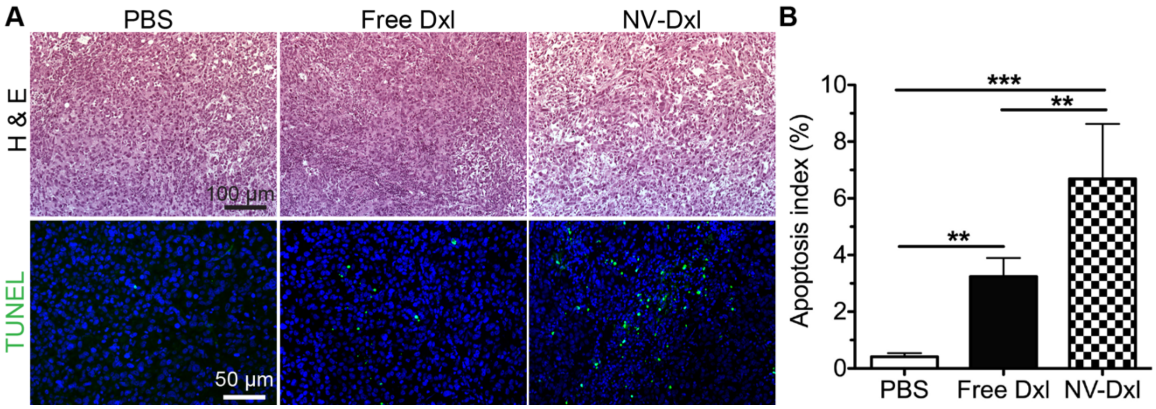

Figure S5. Histology and immunohistochemical analyses of subcutaneous prostate cancer sections.

A: Tumors were stained to examine morphology (H&E) or apoptosis (TUNEL).

B: Apoptosis index was determined as percentage of TUNEL positive cells in 9 randomly chosen 200x fields per group. Data are represented as mean ± SD. **: $p < 0.01$, ***: $p < 0.001$.
